# An FGFR2 mutation as the potential cause of a new phenotype including early-onset osteoporosis and bone fractures: a case report

**DOI:** 10.1186/s12920-023-01750-1

**Published:** 2023-12-14

**Authors:** Ilya S Dantsev, Mariia A Parfenenko, Gulnara M Radzhabova, Ekaterina A Nikolaeva

**Affiliations:** Veltischev Research and Clinical Institute for Pediatrics and Pediatric Surgery of the Pirogov, Russian National Research Medical University of the Ministry of Health of the Russian Federation, 2 Taldomskaya St, Moscow, 125412 Russia

**Keywords:** Osteoporosis, Osteogenesis Imperfecta, BMD, Fracture, *FGFR2*

## Abstract

Osteoporosis is a systemic, multifactorial disorder of bone mineralization. Many factors contributing to the development of osteoporosis have been identified so far, including gender, age, nutrition, lifestyle, exercise, drug use, as well as a range of comorbidities. In addition to environmental and lifestyle factors, molecular genetic factors account for 50–85% of osteoporosis cases. For example, the vitamin D receptor (VDR), collagen type I (COL1), estrogen receptor (ER), apolypoprotein Е (ApoE), bone morphogenetic protein (BMP), and Low-density lipoprotein receptor-related protein 5 (LRP5) are all involved in the pathogenesis of osteoporosis. Among the candidate genes, the pathogenic variants in which are involved in the pathogenesis of osteoporosis is *FGFR2*. Additionally, FGFs/FGFRs-dependent signaling has been shown to regulate skeletal development and has been linked to a plethora of heritable disorders of the musculoskeletal system. In this study we present the clinical, biochemical and radiological findings, as well as results of molecular genetic testing of a 13-year-old male proband with heritable osteoporosis, arthralgia and multiple fractures and a family history of abnormal bone mineralization and fractures. Whole exome sequencing found a heterozygous previously undescribed variant in the *FGFR2* gene (NM_000141.5) (GRCh37.p13 ENSG00000066468.16: g.123298133dup; ENST00000358487.5:c.722dup; ENSP00000351276.5:p.Asn241LysfsTer43). The same variant was found in two affected relatives. These data lead us to believe that the variant in *FGFR2* found in our proband and his relatives could be related to their phenotype. Therefore, modern methods of molecular genetic testing can allow us to differentiate between osteogenesis imperfecta and other bone mineralization disorders.

## Introduction

Osteoporosis is a complex, multifactorial disorder characterized by progressive loss of bone mass and degeneration of bone microarchitecture, which leads to increased bone fragility and risk of fractures [1, 2]. According to research of osteoporosis epidemiology, the frequency of this disorder in 2021 was 18,3% [3]. Osteoporosis has significant detrimental effects on the probands physical health and quality of life, and also requires for increasingly large treatment costs annually. [4]. The understanding of the pathogenetic mechanism of osteoporosis is an important milestone to be reached on the road to its treatment and prevention. Therefore, in addition to environmental and lifestyle factors, molecular genetic factors account for 50–85% of osteoporosis cases [5].

One of the candidate genes, the pathogenic variants in which are involved in the pathogenesis of osteoporosis is *FGFR2*.

Fibroblast growth factor receptor 2 (FGFR2) is a type of receptor tyrosine kinase (RTK) that belongs to the immunoglobulin superfamily [6]. It is expressed mainly in the epithelial and mesenchymal cells and, together with the fibroblast growth factors (FGFs), including FGF1-4, FGF6, 7, plays a key role in the development of, the skeleton and endocrine glands, as well as the skin and several internal organs [7]. The expression of *FGFR2* and the quantity and location of the receptor on the cellular membrane greatly affects the molecular effect of FGFs/FGFR2 signaling [8, 9]. *FGFR2* is known to regulate the development of osteoblasts. Increased FGFs/FGFRs-dependent signaling can lead to hyperplasia of immature osteoblasts, inhibit their differentiation and trigger apoptosis [10]. The imbalance between the rate of osteosynthesis, regulated by osteoblasts, and bore resorption, regulated by osteoclasts, is believed to be the main mechanism of osteoporosis. Recent findings suggest that *FGFR2* also regulates the development of chondrocytes [11]. However, the role of *FGFR2* in the pathogenetic mechanism of osteoporosis needs further research.

In this study we present the clinical, biochemical and radiological findings, as well as results of molecular genetic testing of a 13 year old male proband that presented with multiple fractures, decreased tolerance of physical activity, arthralgia in the legs, spine and joints, severe dental caries, decreased density of dental enamel and frequent headaches. Based on the clinical and laboratory findings, the original diagnosis was osteogenesis imperfecta type I. However, whole exome sequencing found a single previously undescribed variant in the *FGFR2* gene.

## The case report

### Anamnesis vitae

**Fig. 1:**
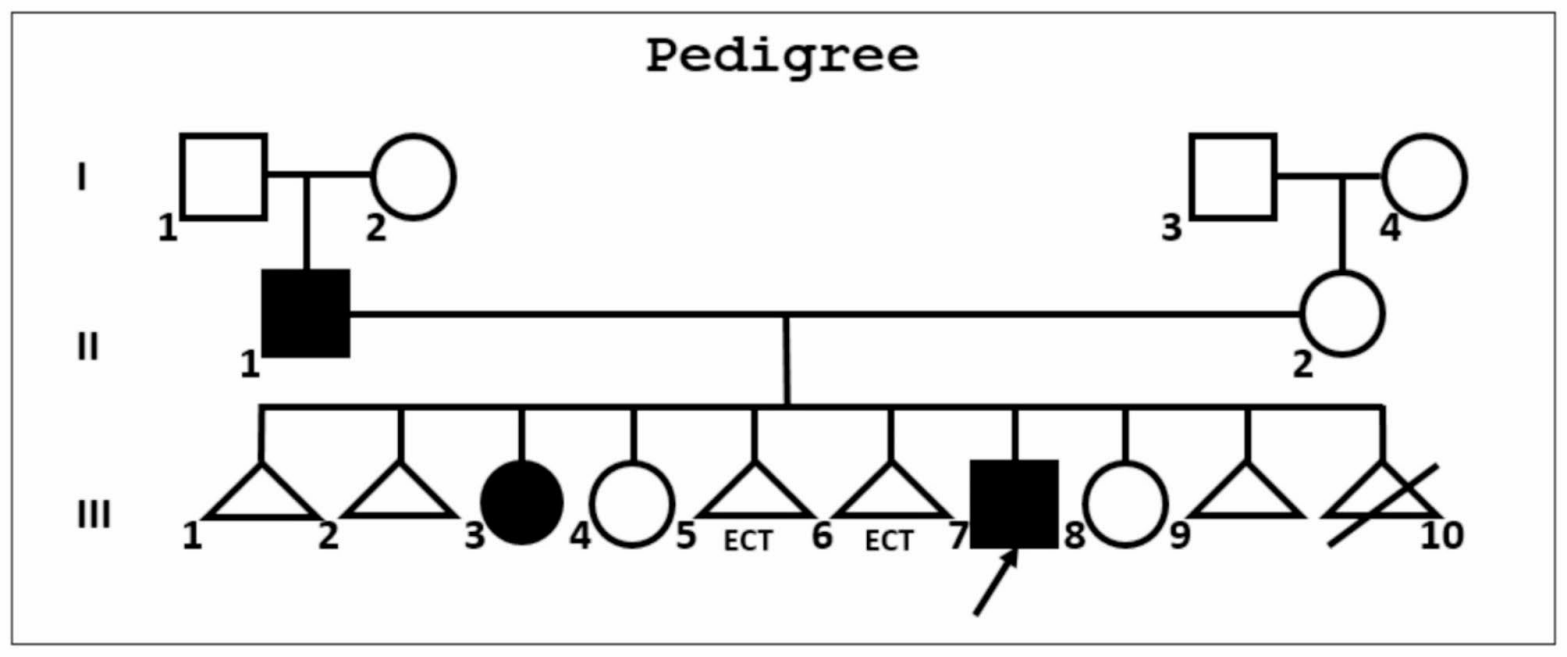
A circle indicates a female, a square – a male. A horizontal line indicates marriage, while vertical lines united by a horizontal line indicates children born from the same set of parents. A triangle indicates a prenatal death. A colored shape indicates an affected individual. The proband’s immediate family: II-1 – male (had fractures in childhood); II-2 – female, healthy; III-1-2 silent miscarriage in the second trimester; III-3 - female, born in 2003 that has the following phenotypic features: genu valgum, hip dysplasia, combined thoracolumbar scoliosis, connective tissue disorder; III-4 - female, born in 2005, healthy; III-5-6 – ectopic pregnancy; III-7 – proband; III-8 – female, born in 2014, healthy. III-9 – miscarriage in the first trimester; III-10 – medical abortion

A 13 year old male born was as result of the VII pregnancy, from unrelated parents. Other pregnancies resulted in: I-II silent miscarriage in the second trimester; III - female, born in 2003 (III-3 Fig. 1) that has the following phenotypic features: *genu valgum*, hip dysplasia, combined thoracolumbar scoliosis, connective tissue disorder; IV - female, born in 2005 (III-4 Fig. 1), healthy; V-VI – ectopic pregnancy; VIII – female, born in 2014 (III-5 Fig. 1), healthy. IX – miscarriage in the first trimester; X – medical abortion. The course of pregnancy with the proband was uncomplicated apart from toxicosis in the first trimester. Childbirth occurred at 37 weeks of gestation and was natural. Birth weight of the proband was 2750 g., and birth length − 55 sm. His Apgar score − 8–9 points. The main milestones in his early psychomotor development were reached at appropriate ages: held his head up since 1 month of age, sat since 7 months of age, walked since 12 months of age, first words appeared at 12 months of age, dental eruption - at 6 months of age.

### Anamnesis Morbi

The first bone fracture (distal phalange of the second finger of the right hand) occurred at the age of 1. At the age of 3, the proband fractured his nasal bones, and then fractured them again at 4 years old. At the age of 7, he fractured the left ulna and radius. At the age of 9 he fractured his fibula. Since the age of 10 – numerous fractures of the phalanges of the feet and hands: at 11 – fracture of the first toe of the right foot, at 12 - fracture of the fith toe of the right foot and the distal phalange of the second finger of the right hand. The last documented fracture (3,4 metacarpal bones of the right hand) occurred in January 2022. All fractures resulted from low-impact trauma.

In 2019, the proband consulted a geneticist at the Research center for medical genetics, who suggested the presumptive diagnosis of osteogenesis imperfecta type I. Molecular genetic testing using the massively parallel signature sequencing method that involved the analysis of the 17 genes currently associated with osteogenesis imperfecta (*BMP1*, *COL1A1*, *COL1A2*, *CREB3L1*, *CRTAP*, *FKBP10*, *IFITM5*, *LEPRE1*, *PLOD2*, *PLS3*, *PPIB*, *SERPINF1*, *SERPINH1*, *SP7*, *SPARC*, *TMEM38B*, *WNT1*) showed no pathogenic variants.

**Fig. 2:**
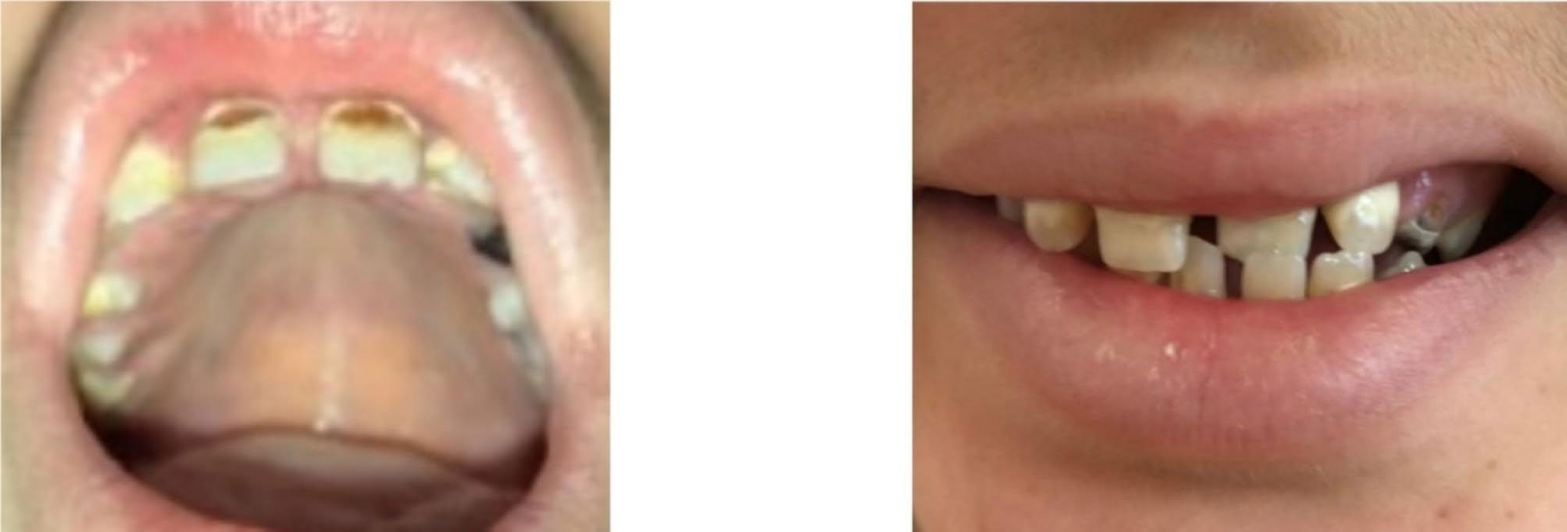
The probands (13 years old) teeth. Severe dental caries, decreased density of dental enamel

In order to achieve the correct diagnosis and determine the prognosis for the disease progression, the proband was hospitalized to the genetics department of the Veltischev Institute in 2020, at the age of 11. The physical examination showed: scoliosis, *genu valgum*, flexion contracture of the ankles, *pes planus*, severe dental caries, decreased density of dental enamel (Fig. 2). The proband complains of multiple fractures, decreased tolerance of physical activity, arthralgia in the legs, spine and joints, enamel and frequent headaches. The proband had normal intelligence.

### Results of instrumental and laboratory diagnostic procedures

**Fig. 3:**
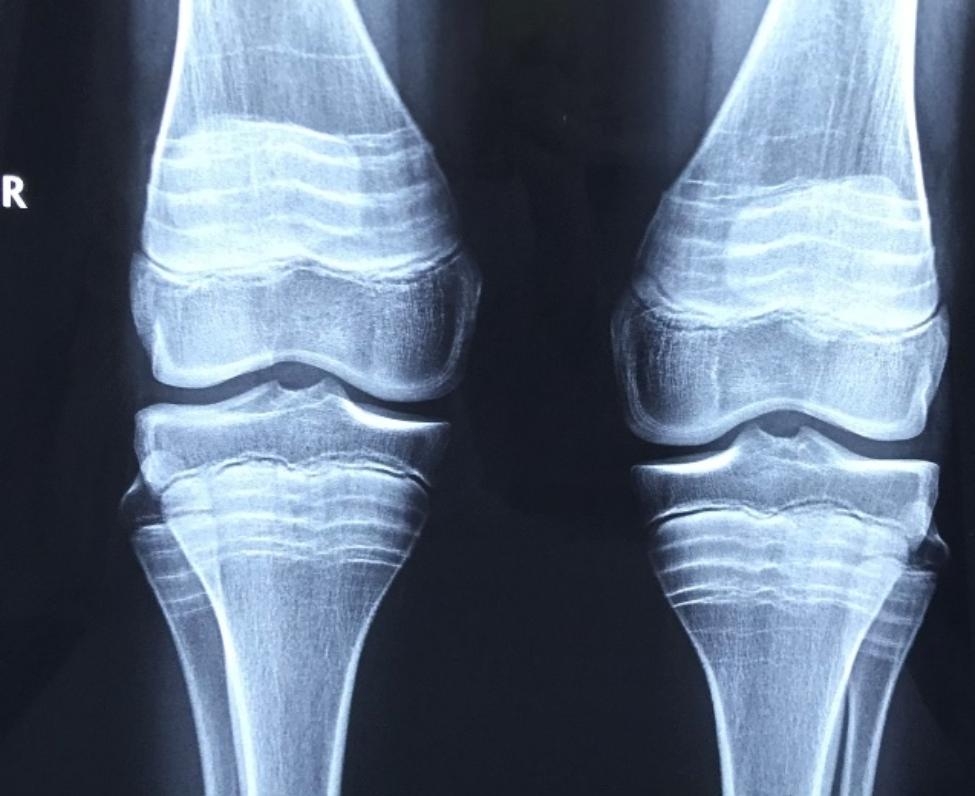
The radiograph of the knee joints of the proband (13 years old): the metaphyses are bell-shaped expanded, with transverse bands of sclerosis - likely resulting from intravenous bisphosphonates. The cortical layer is thinned. The structure of the bone tissue is sparse. The general structure of the joint is intact. The articular surfaces are congruent. Articular spaces are symmetrical. Growth zones are well defined

Instrumental methods of analysis showed results suggestive of a bone mineralization disorder. Bone mineral density measurements performed on GE MS LUNAR Prodigy densitometer with the protocol AP Spine showed a Z-score= -2,3, and with the protocol Total body: Z-score= -1,6. The radiograph of the knee joints in a direct projection (Fig. 3) confirms *genu valgum*. The metaphyses are bell-shaped expanded, with transverse bands of sclerosis. The cortical layer is thinned. The structure of the bone tissue is sparse. The general structure of the joint is intact. The articular surfaces are congruent. Articular spaces are symmetrical. Growth zones are well defined. The main biochemical markers of bone metabolism: parathyroid hormone: 72 pg/ml (N 16.0–62.0); bone alkaline phosphatase: 48,91 mcg/l (N 48.06–120.0); 25–25-hydroxyvitamin D: 14,1 ng/ml (N 14.0–60.0); Calcium (ionized) 1.15 mmol/l (N 1,13 − 1,32); Calcium (total) 2.58 mmol/l (N 2,20 − 2,65); Phosphorus (inorganic) 1.66 mmol/l (N 1,29 − 2,26); Ostase 23.86 mcg/l (N 62,05–120,00).

Biochemical analysis of 24 h urine: Са/Crea 0.09 (N 0,04 − 0,70), Ph/Crea 2.02 (N 0,80 − 2,70). Rheumatoid factors were normal.

Taking into consideration the clinical data and presumptive diagnosis, the decision was made to start the proband on zoledronic acid and recommending whole exome sequencing in order to determine the underlying genetic cause.

Whole exome sequencing (WES) was carried out using Illumina SureSelect All Exon V7 with average depth across the target regions of > 10X, and analyzed using an automated algorithm, that allowed quality control of sequencing (FASTQC tool), trimming of adapter sequences from short read data (SEQPURGE tool), mapping low-divergent sequences (BWA MEM tool), marking duplicates and extracting discordant and split reads from sam files (SAMBLASTER tool), increased accuracy of localized assembly and global realignment (ABRA2 tool), haplotype-based variant detector (FREEBAYES tool), and variant effect predictions (ENSEMBL-VEP tool). The algorithm was tested on pre interpreted exome date from “Genome in a bottle”. The sensitivity of the algorithm is 98,6% and the specificity – 99.1%. None of the authors directly participated in WES.

The variants were filtered in order not to include those that have a population frequency of > 10%, as well as those that have no impact on the resulting protein structure. Each variant localized in genes that were potentially related to the probands phenotype was viewed in regard to it’s effect on the protein structure and function, as well as evolutionary conservatism of the position within the gene, the clinical features of the disorder associated with the gene, as well as the population frequency of the variant and the inheritance pattern of the related disorder. The variants were classified into 5 categories: (pathogenic, likely pathogenic, variant of uncertain significance, likely benign and benign), according to ACMG/AMP 2015 guideline.

**Fig. 4:**
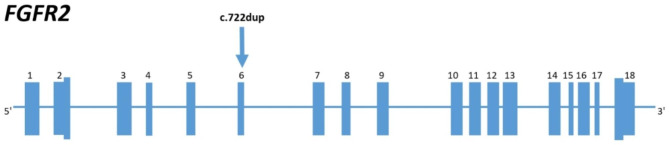
The location of the variant in the *FGFR2* gene

WES showed a previously undescribed heterozygous variant in exon 6 of 18 of the gene *FGFR2*, which consists of a single nucleotide insertion and leads to a frameshift and a premature translation termination (NM_000141.5) (GRCh37.p13 ENSG00000066468.16: g.123298133dup; ENST00000358487.5:c.722dup; ENSP00000351276.5:p.Asn241LysfsTer43) (Fig. 4). No other potentially clinically significant variants in genes associated with bone mineralization were found.

The variant is not present in the gnomAD population databases and likely leads to loss-of-function. According to OMIM, pathogenic variants in *FGFR2* can cause autosomal dominant Antley-Bixler syndrome without genital anomalies or disordered steroidogenesis (OMIM: 207410), as well as Beare-Stevenson cutis gyrata syndrome (OMIM: 123790), Apert syndrome (OMIM: 101200), Bent bone dysplasia syndrome (OMIM: 614592) and other conditions, all of which significantly differ from the proband’s phenotype. Meanwhile, there is data available that suggests a relationship between pathogenic variants in *FGFR2* and a disorder of bone mineralization that leads to fractures of both small and large tubular bones [12].

**Fig. 5:**
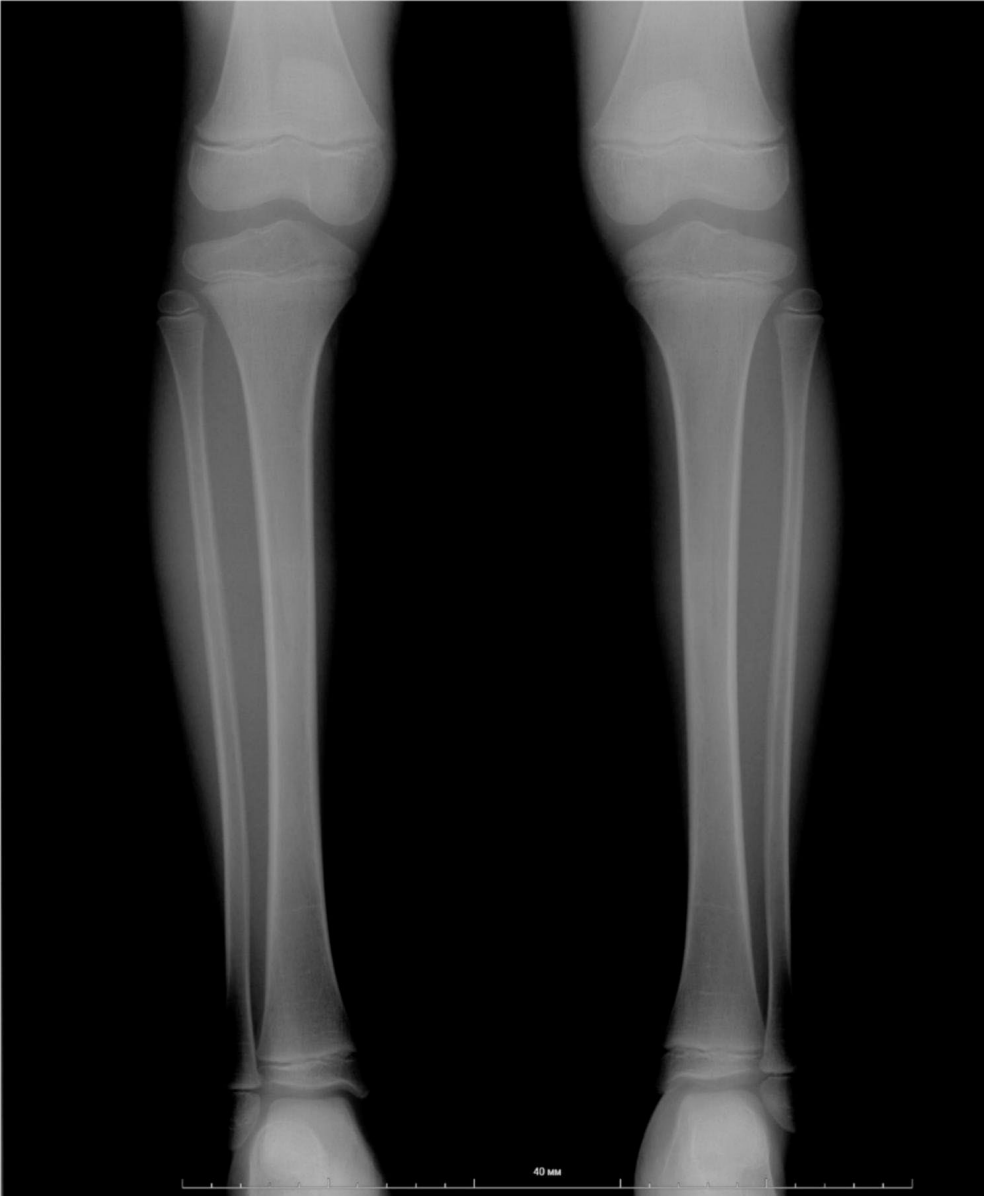
The radiograph of the legs of the probands sister (7 years old) in a direct projection: genu valgum and a mild decrease in bone density

In 2021, analysis of the variant segregation in the family was conducted using Sanger sequencing. The same variant (NM_000141.5). (GRCh37.p13 ENSG00000066468.16: g.123298133dup; ENST00000358487.5:c.722dup; ENSP00000351276.5:p.Asn241LysfsTer43) was found in the proband’s father, who has had 3 fractures in childhood, two of which resulted from low-impact trauma. He, however, has had neither high-impact trauma, nor fractures in adulthood. The same variant was also observed in the proband’s sister, who presented with *genu valgum*, hip dysplasia, combined thoracolumbar scoliosis, and a connective tissue disorder. She also had severe dental caries and decreased density of dental enamel. Bone mineral density measurements performed on GE MS LUNAR Prodigy densitometer with the protocol AP Spine showed a Z-score = -1,5, and with the protocol Total body - Z-score = -1,4. The radiograph of the sister’s legs in a direct projection (Fig. 5) confirms *genu valgum* and shows a mild decrease in bone density. She, however, did not have any bone fractures to date. Other family members didn’t have the variant.

The variant (NM_000141.5) (GRCh37.p13 ENSG00000066468.16: g.123298133dup; ENST00000358487.5:c.722dup; ENSP00000351276.5:p.Asn241LysfsTer43) has been classified as pathogenic according to the ACMG/AMP 2015 guideline, with consideration of the recommendations for interpretation of the null variant criteria (PVS1) provided by Abou Tayoun AN et al. [13] based on three criteria: PM2: Absent from controls (or at extremely low frequency if recessive), PP1: Co segregation with disease in multiple affected family members, and PVS1: null variant (nonsense, frameshift, canonical ± 1 or 2 splice sites, initiation codon, single or multi-exon deletion) in a gene where loss-of-function is a known mechanism of disease.

Since the variant is located in exon 6 of 18 and leads to premature translation termination, it is highly likely that the polypeptide chain synthesized from the allele containing the variant undergoes nonsense mediated decay. However, the “in vivo” or “in vitro” validation of this hypothesis has not yet been carried out.

Some genes may tolerate loss-of-function (LOF) variants because their functional effects are masked by incomplete penetrance [14], by compensatory variants [15], or because of a low functional impact of the truncation [16], this is why other methods of molecular genetic testing, such as functional analysis may be required to determine the effect of the observed variant, further classifying it as either pathogenic or benign.

At the moment of publication of this case report, the proband has had four courses of treatment with zoledronic acid. During the period between hospitalizations, the proband took chondroprotectors (Osteogenon), colecalciferol, and maintained a program of physical therapy and exercise. During another hospitalization in the spring of 2022, we observed significant improvement. Bone mineral density measurements performed on GE MS LUNAR Prodigy densitometer with the protocol AP Spine showed a Z-score L1-L4 = -0,2, and with the protocol Total body: Z-score = -0,9, which is 7% lower than the population norm А/G = 0,8. The main biochemical markers of bone metabolism and rheumatoid factors were normal. The proband’s legal guardians gave consent to the aforementioned procedures, including the molecular genetic testing.

## Discussion

In this case report we describe a proband with clinical features of osteogenesis imperfecta, which was later disproven by molecular genetic testing. Whole exome sequencing showed a variant in the *FGFR2* gene (NM_000141.5) (GRCh37.p13 ENSG00000066468.16: g.123298133dup; ENST00000358487.5:c.722dup; ENSP00000351276.5:p.Asn241LysfsTer43) that likely leads to its loss-of-function. This gene has a major role in bone tissue development. This, combined with the results of the analysis of segregation of the variant in the probands family, that showed the same variant was present in the father and sister both of whom presented bone mineralization disorders (the father has had multiple bone fractures in childhood and the sister presents with *genu valgum*, hip dysplasia, combined thoracolumbar scoliosis and decreased signs of osteoporosis according to the densitometry), leads us to believe that the variant in *FGFR2* found in our proband and his relatives is related to their phenotype. However, the functional analysis of the gene has not been carried out at the time of publication, and the currently available data can only indirectly explain role of a loss-of-function of *FGFR2* in FGFs/FGFRs-dependent signaling and how it could result in a bone mineralization disorder with highly variable clinical features among several affected individuals within one family.

It is known that gain of function (GOF) mutations in *FGFR2*, primarily in the III Ig-like domain and the adjacent linker regions (exons IIIa and IIIc), can cause several different types of autosomal dominant craniosynostosis, including Crouzon syndrome, Pfeiffer syndrome, and Apert syndrome [17]. One study specifically described several de novo missense mutations in *FGFR2* that for perinatal lethal skeletal dysplasia, termed the BBDS-FGFR2 type, characterized by multiple bone deformities, including impaired mineralization of the calvarium, craniosynostosis, and dysmorphic facial features, as well as long bone deformities and osteopenia [18].

The FGF family includes 23 different genes, and the human FGFR consists of FGFR1-5, so the disruption of each of the components can play a major role in the physiological and pathological processes regulated by FGFs/FGFRs-dependent signaling. *FGFR1* and *FGFR2* are expressed by mesenchymal cells before the appearance of the morphological signs of mesenchemal condensation [19]. *FGFR1* is evenly expressed in the mesenchyme of limb buds, while *FGFR2* expression is increased in the area of ​​chondrogenic condensation and is typically the first observable biochemical marker of this process. Both *FGFR1* and *FGFR2* are expressed at the periphery of the indurations, where the original cells of the perichondrium and periosteum are found. It was also found that *FGFR2* is expressed in the resting zone, while *FGFR4* is expressed in both the resting and proliferative zones. *FGFR3* is more actively expressed in latent chondroprogenitor cells located in Ranvier’s sulcus and LaCroix’s annulus. The patterns of expression of *FGFR1, FGFR2* and *FGFR3* are relatively well described in literature. In cranial sutures, FGFRs expression in spatially dependent. *FGFR2* is predominantly expressed in osteoprogenitor cells, while *FGFR1* - in more differentiated osteoblasts. *FGFR3* has a lower expression in the periosteum and suture osteogenic fronts at a late stage of suture development. The binging of FGF2 and FGFR1 on the cellular membrane of osteoclasts can activate MAPK signaling pathway, therefore regulating their differentiation and function [20]. Additionally, FGFR3, together with FGF2 regulate osteoclast function via the ERK signaling pathway. FGF23 is known to regulate osteoblast function [21], and Wei at al showed that hyperfunction of FGF21 decreased bone density on animal models [22]. A growing body of evidence suggests that FGFs/FGFRs-dependent signaling plays a critical role in numerous processes during embryonic development, as well as affects adult homeostasis.

Therefore, having analyzed the case of early-onset osteoporosis with bone fractures, as well as available scientific data, we consider it likely that pathogenic variants in *FGFR2* can lead to the development of a bone mineralization disorder, specifically associated with an earlier age of onset.

## Conclusion

Currently, the role of numerous molecular genetic factors in the pathogenesis of osteoporosis and other bone mineralization disorders requires further research. Deeper understanding of this problem can bring us closer to understanding their underlying mechanism and help differentiate between heritable osteoporosis, osteogenesis imperfecta and other clinically similar conditions. And early diagnosis allows for development of a more effective therapeutic strategy and rehabilitation program for probands with bone mineralization disorders, as well as decrease the risk of complications, such as fractures and arthralgia.

The available clinical and genetic data indicate that pathogenic variants in the *FGFR2* gene could be linked to a new phenotype of early onset osteoporosis with bone deformities and fractures.

## Data Availability

The datasets supporting the conclusions of this article are included within the article. The raw sequencing data is available in the Sequence Read Archive (SRA) submission: PRJNA997484.
